# U.S. House Prices by Census Division: Persistence, Trends and Structural Breaks

**DOI:** 10.1007/s11294-023-09868-9

**Published:** 2023-03-20

**Authors:** Guglielmo Maria Caporale, Luis Alberiko Gil-Alana

**Affiliations:** 1 Brunel University, London, UK; 2 University of Navarra, Pamplona, Spain; 3 Department of Economics, Universidad Francisco de Vitoria, Madrid, Spain

**Keywords:** U.S. house prices, Fractional integration, Persistence, Trends, Structural breaks, C22, E30

## Abstract

This paper uses fractional integration methods to examine persistence, trends and structural breaks in United States house prices, more specifically the monthly Federal Housing Finance Agency House Price Index for census divisions, and the United States as a whole over the period from January 1991 to August 2022. The full sample estimates imply that the order of integration of the series is above one in all cases, and is particularly high for the aggregate series, implying high levels of persistence. However, when the possibility of structural breaks is taken into account, segmented trends are detected. The subsample estimates of the fractional differencing parameter tend to be lower, with mean reversion occurring in a number of cases. This means that shocks in the series are expected to be transitory in these subsamples, disappearing in the long run by themselves. In addition, the time trend coefficient is at its highest in the last subsample, which in most cases starts around May 2020 coincident with the beginning of the coronavirus pandemic. The results provide clear evidence of differences between census divisions, which implies that appropriate housing policies should be designed at the local (rather than at the federal) level.

## Introduction

House prices are an important factor affecting the real economy as well as financial markets. Their key importance was shown very clearly by the 2007 sub-prime mortgage crisis in the U.S., which was mainly caused by a housing bubble that started in the previous decade (Shiller, 2007). The empirical literature aiming to shed light on their behaviour comprises two main strands. The first type of studies analyses their relationship with economic fundamentals. For instance, Capozza and Helsely (1989, 1990) provided evidence on the long-run equilibrium relationship between real house prices and real income.

The second category of papers focuses instead on the univariate statistical properties of house prices. Earlier studies carried out unit root tests (e.g., Meen, 1999, for United Kingdom (UK) regional prices; Cook & Vougas, 2009 for aggregate prices in the presence of structural breaks; Clark & Coggin, 2011, and Zhang et al., 2017, for the U.S.; Arestis & Gonzales, 2014, for 18 Organization for Economic Co-operation and Development (OECD) countries). However, this approach, which is based on the dichotomy between I(0) stationary and I(1) non-stationary processes, imposes rather restrictive assumptions on the behaviour of the series of interest. A more general framework, which is more informative about properties such as persistence and mean reversion, is represented by fractional integration (Granger, 1980; Granger & Joyeux, 1980; Hosking, 1981). In this case, the series of interest is modelled as an I(*d*) process, with *d* being allowed to take any real value, including fractional ones. This approach encompasses a wide range of stochastic behaviours. More specifically, if *d* = 0, the process is said to exhibit short memory, with its autocorrelations (if non-zero) decaying at an exponential rate. If *d* > 0, the process is characterised by long memory, and the autocorrelations decay at a rather slower hyperbolic rate. If 0 < *d* < 0.5, the process is covariance stationary and, as long as *d* < 1, mean reversion will occur, even if the fractional parameter is in the non-stationary range. Finally, *d* = 1 corresponds to the unit root case, and *d* > 1 to explosive behaviour. Papers modelling house prices using this method include among others Gupta et al. (2014). However, all these studies focus on long-run persistence only and do not allow for possible breaks. More recently, Canarella et al. (2021) instead used a fractional integration model including both a long-run and a cyclical component to analyse persistence in both U.S. and UK house prices over a long time span, and also tested for breaks. They found that long-run persistence plays a greater role. Breaks occurred at different times in the two countries being examined (earlier in the U.S.), which implies that national factors were their main drivers of house prices.

The present study belongs to the second strand of the literature on house prices, which carries out univariate analysis, and also follows a fractional integration approach as in the more recent contributions mentioned previously. However, unlike them, the present study provides evidence on U.S. house price behaviour by geographical area. More specifically, it examines data for various census divisions. This is an important addition to the existing body of empirical literature, since there can obviously be significant differences between the housing markets of different areas of a country (the U.S., in the current case) that are not captured by the aggregate price series. Thus, different policy prescriptions might be appropriate in each case. The other issue addressed by the current analysis is the possible presence of breaks in the series under examination, which is also of key importance to understand changes in the housing market that might have occurred as a result of a variety of factors (fundamentals or others), again with implications for the design of effective stabilisation policies.

## Data Description and Modelling Framework

Monthly Federal Housing Finance Agency (FHFA) House Price Index data for census divisions and the U.S. as a whole were analyzed. The census divisions are East North Central, East South Central, Middle Atlantic, Mountain, New England, Pacific, South Atlantic, West North Central, West South Central. The sample period is January 1991 to August 2022. The series are not seasonally adjusted and were obtained from the House Price Index Datasets of the Federal Housing Finance Agency (2023).

The model is specified as follows:

1 $$\begin{array}{ccc}{y}_{t}=\alpha +\beta t+{x}_{t},& {\left(1-B\right)}^{d}{x}_{t}={u}_{t},& {u}_{t}=\rho {u}_{t-12}+{\varepsilon }_{t}\end{array}$$

where *y*_*t*_ stands for the series of interest, *α* and *β* denote the constant and the coefficient on a linear time trend, respectively, *B* is the backshift operator, i.e., *Bx*_*t*_ = *x*_*t-1*_*,* and *u*_*t*_ is a short-memory process which is integrated of order 0. Note that* d* is allowed to take any real value, including fractional ones. Thus, as already mentioned, the chosen framework encompasses a wide range of specifications, such as the classical trend stationary I(0) model if *d* = 0, the unit root case if *d* = 1, anti-persistence if *d* < 0, and long memory if *d* is positive and has a fractional value. In the latter case, if 0 < *d* < 0.5, the series is still covariance stationary. If *d* < 1, mean reversion occurs.

## Empirical Results

First, the values of *d* were estimated from the model given by Eq. (1) under the assumption that the error term, *u*_*t*_*,* is a white noise process. Following the standard literature on unit roots (e,g., Bhargava, 1986; Schmidt & Phillips, 1992), three specifications were considered, including respectively: (i) no deterministic terms, i.e. *α* = *β* = 0; (ii) a constant only, i.e. *β* = 0; and (iii) both a constant and a linear time trend, i.e. *α* ≠ 0 and *β* ≠ 0. The selected specification was chosen by looking at the t-values of the estimated coefficients for these deterministic terms. The results for each series are displayed in Table 1 (upper part for the original data and lower part for the log-transformed data). Starting with the original data (Table 1, upper level), it can be seen that the coefficient on the time trend is significant for six out of the nine census divisions examined (i.e., in all cases except Mountain, Pacific and South Atlantic), but is insignificant for the U.S. aggregate data. The largest values on the time trend are for the West South Central (0.761) and East South Central (0.758). The estimated values of *d* are significantly higher than one in all cases, ranging from 1.24 (East South Central) and 1.25 (West North Central) to 1.53 (Mountain) and 1.55 (Pacific). For the U.S. aggregate data, the time trend is insignificant and the order of integration is 1.70, much higher than for the individual census divisions, which is probably due to the aggregation effect on the degree of integration of the series (Granger, 1980; Robinson, 1978). Focussing now on the log-transformed data (Table 1, lower level), the time trend is now significantly positive in all cases except for the Pacific division and for the aggregate data. The estimates of d are slightly smaller than before (between 1.13 for East South Central and 1.45 for Pacific), and again higher for the aggregate series (*d* = 1.52), the unit root null hypothesis being rejected in all cases in favour of *d* > 1. In other words, mean reversion does not occur in any single case, and thus shocks have permanent effects.

**Table 1:** Estimated coefficients white noise disturbances

Original data			
Series (original)	No terms	An intercept	An intercept and a linear time trend
EAST NORTH CENTRAL	1.34 (1.30, 1.39)	99.407 (121.43)	0.593 (2.21)
EAST SOUTH CENTRAL	1.24 (1.20, 1.28)	99.430 (78.24)	0.758 (3.07)
MIDDLE ATLANTIC	1.31 (1.26, 1.36)	99.668 (96.34)	0.563 (1.93)
MOUNTAIN	1.53 (1.46, 1.62)	100.274 (61.51)	–
NEW ENGLAND	1.26 (1.22, 1.31)	99.278 (67.62)	0.715 (2.25)
PACIFIC	1.55 (1.50, 1.62)	99.839 (82.55)	–
SOUTH ATLANTIC	1.43 (1.38, 1.49)	99.831 (87.45)	–
WEST NORTH CENTRAL	1.25 (1.21, 1.29)	99.482 (90.00)	0.734 (3.24)
WEST SOUTH CENTRAL	1.32 (1.27, 1.37)	99.620 (92.60)	0.761 (2.39)
USA	1.70 (1.61, 1.80)	99.808 (174.67)	–
Log-transformed data			
Series (in logs)	No terms	An intercept	An intercept and a linear time trend
EAST NORTH CENTRAL	1.25 (1.22, 1.30)	4.601 (1022.48)	0.0033 (3.62)
EAST SOUTH CENTRAL	1.13 (1.09, 1.17)	4.601 (741.05)	0.0036 (5.48)
MIDDLE ATLANTIC	1.24 (1.20, 1.29)	4.603 (867.52)	0.0030 (2.91)
MOUNTAIN	1.36 (1.31, 1.42)	4.604 (759.88)	0.0042 (1.93)
NEW ENGLAND	1.20 (1.16, 1.24)	4.600 (626.40)	0.0034 (2.96)
PACIFIC	1.45 (1.41, 1.50)	4.603 (853.90)	–
SOUTH ATLANTIC	1.34 (1.30, 1.39)	4.601 (866.00)	0.0036 (2.20)
WEST NORTH CENTRAL	1.16 (1.12, 1.20)	4.602 (895.61)	0.0036 (5.55)
WEST SOUTH CENTRAL	1.17 (1.13, 1.22)	4.602 (968.32)	0.0037 (5.96)
USA	1.52 (1.47, 1.59)	4.603 (1685.17)	–

In brackets are the 95% confidence bands in column 2, and the t-statistics for the estimated coefficients in columns 3 and 4. In all cases, the *p*-values were < 0.005. The starting date is January 1991 and the final date date is August 2022. The series were obtained from the House Price Index Datasets of the Federal Housing Finance Agency (2023)

The results considered so far might be biased owing to the strong assumption that the residuals are a white noise process. Thus, in what follows, autocorrelation is allowed for. In particular, rather than imposing a parametric autoregressive moving average (ARMA) model that would require specifying the correct AR and MA orders (which is not straightforward in the context of fractional integration, Beran et al., 1998) the non-parametric modelling approach of Bloomfield (1973) was applied, which is based on a spectral density function, whose log form approximates well that of AR structures. Table 2 reports the corresponding results for the original and log-transformed data in the upper and lower parts, respectively. The time trend is now insignificant in every single case, the intercept being the only deterministic term required in the model. As for *d*, its estimated values are again significantly higher than one in all cases, ranging from 1.41 (New England, logged values) to 2.03 (Pacific, original data).

**Table 2:** Estimated coefficients using the original series. Bloomfield disturbances

Original data			
Series (original)	No terms	An intercept	An intercept and a linear time trend
EAST NORTH CENTRAL	1.51 (1.42, 1.64)	99.641 (136.09)	–
EAST SOUTH CENTRAL	1.59 (1.50, 1.73)	99.765 (98.64)	–
MIDDLE ATLANTIC	1.56 (1.46, 1.78)	99.914 (110.86)	–
MOUNTAIN	1.90 (1.55, 2.34)	100.53 (73.15)	–
NEW ENGLAND	1.45 (1.38, 1.55)	99.385 (75.91)	–
PACIFIC	2.03 (1.82, 2.23)	99.722 (98.56)	–
SOUTH ATLANTIC	1.65 (1.52, 1.90)	99.791 (98.56)	–
WEST NORTH CENTRAL	1.49 (1.41, 1.61)	99.840 (105.16)	–
WEST SOUTH CENTRAL	1.51 (1.41, 1.65)	100.08 (105.26)	–
USA	1.63 (1.51, 1.91)	99.818 (169.44)	–
Log transformed data			
Series (in logs)	No terms	An intercept	An intercept and a linear time trend
EAST NORTH CENTRAL	1.46 (1.39, 1.55)	4.601 (1143.88)	–
EAST SOUTH CENTRAL	1.48 (1.40, 1.56)	4.601 (865.95)	–
MIDDLE ATLANTIC	1.53 (1.45, 1.66)	4.604 (1036.23)	–
MOUNTAIN	1.67 (1.53, 1.86)	4.608 (898.17)	–
NEW ENGLAND	1.41 (1.34, 1.50)	4.599 (702.80)	–
PACIFIC	1.94 (1.80, 2.09)	4.602 (1155.50)	–
SOUTH ATLANTIC	1.60 (1.51, 1.73)	4.603 (1015.44)	–
WEST NORTH CENTRAL	1.45 (1.38, 1.55)	4.603 (1030.14)	–
WEST SOUTH CENTRAL	1.45 (1.36, 1.57)	4.605 (1118.43)	–
USA	1.62 (1.51, 1.76)	4.603 (1771.70)	–

In brackets are the 95% confidence bands in column 2, and the t-statistics for the estimated coefficients in columns 3 and 4. In all cases, the *p*-values were < 0.005. The starting date is January 1991 and the final date is August 2022. The series were obtained from the House Price Index Datasets of the Federal Housing Finance Agency (2023)

Finally, given the monthly frequency of the data, a seasonal AR(1) process in the error term was allowed for. As reported in Table 3, the results are very similar to the previous ones obtained under the assumption of white noise errors. The time trend is not required for the Mountain, Pacific, and South Atlantic divisions or for the aggregate data (U.S.), and the degrees of integration are higher than one in all cases, their estimated values being larger for the original data compared to the log-transformed data.

**Table 3:** Estimated coefficients. Seasonal AR(1) disturbances

Original data			
Series (original)	No terms	An intercept	An intercept and a linear time trend
EAST NORTH CENTRAL	1.38 (1.33, 1.43)	99.401 (121.5)	0.582 (1.79)
EAST SOUTH CENTRAL	1.24 (1.20, 1.28)	99.430 (78.24)	0.785 (3.07)
MIDDLE ATLANTIC	1.33 (1.28, 1.39)	99.676 (96.68)	0.545 (1.79)
MOUNTAIN	1.56 (1.48, 1.65)	100.298 (62.24)	–
NEW ENGLAND	1.26 (1.22, 1.32)	99.278 (67.62)	0.715 (2.25)
PACIFIC	1.57 (1.52, 1.64)		–
SOUTH ATLANTIC	1.45 (1.40, 1.51)	99.834 (83.20)	–
WEST NORTH CENTRAL	1.26 (1.22, 1.31)	99.845 (90.19)	0.731 (3.06)
WEST SOUTH CENTRAL	1.33 (1.29, 1.38)	99.630 (92.79)	0.751 (2.24)
USA	1.63 (1.51, 1.91)	99.818 (169.44)	–
Log-transformed data			
Series (in logs)	No terms	An intercept	An intercept and a linear time trend
EAST NORTH CENTRAL	1.29 (1.25, 1.34)	4.600 (1029.49)	0.0034 (3.01)
EAST SOUTH CENTRAL	1.13 (1.09, 1.17)	4.601 (741.00)	0.0036 (5.48)
MIDDLE ATLANTIC	1.26 (1.22, 1.31)	4.603 (870.90)	0.0029 (2.57)
MOUNTAIN	1.38 (1.33, 1.44)	4.604 (761.84)	0.0041 (1.78)
NEW ENGLAND	1.20 (1.15, 1.25)	4.600 (626.04)	0.0034 (2.96)
PACIFIC	1.47 (1.42, 1.52)	4.603 (856.91)	–
SOUTH ATLANTIC	1.35 (1.31, 1.40)	4.601 (869.11)	0.0036 (1.98)
WEST NORTH CENTRAL	1.18 (1.14, 1.22)	4.602 (898.31)	0.0035 (4.99)
WEST SOUTH CENTRAL	1.19 (1.15, 1.24)	4.602 (971.82)	0.0037 (5.32)
USA	1.61 (1.51, 1.76)	4.603 (1756.65)	–

In brackets are the 95% confidence bands in column 2, and the t-statistics for the estimated coefficients in columns 3 and 4. In all cases, the *p*-values were < 0.005. The starting date is January 1991 and the final date is August 2022. The series were obtained from the House Price Index Datasets of the Federal Housing Finance Agency (2023)

The high degree of persistence implied by the estimated values of *d* reported in Tables 1, 2, and 3 might be the result of misspecification due to the presence of structural breaks that have not been taken into account. In fact, given the long timespan covered by the data, this is most likely to have occurred. Therefore, Bai and Perron (2003) tests for multiple breaks were conducted as well as the version proposed for the fractional integration case (Gil-Alana, 2008). The break dates detected by means of these two sets of tests were identical and are displayed in Table 4.

**Table 4:** Structural breaks in the series

Series (original)	N. of breaks	Break dates
EAST NORTH CENTRAL	3	April 2006; October 2011; May 2020
EAST SOUTH CENTRAL	4	November 2004; April 2007; October 2011; May 2020
MIDDLE ATLANTIC	4	June 1999; May 2007; February 2012; May 2020
MOUNTAIN	4	September 2003; February 2007; June 2011; June 2020
NEW ENGLAND	4	January 1999; November 2005; January 2012; May 2020
PACIFIC	5	February 1997; October 2003; October 2006; February 2012; May 2020
SOUTH ATLANTIC	4	January 1998; April 2007; July 2011; May 2020
WEST NORTH CENTRAL	3	June 2007; April 2011; May 2020
WEST SOUTH CENTRAL	2	July 2011; June 2020
USA	4	January 1998; April 2007; August 2011; May 2020

The starting date is January 1991 and the final date is August 2022. The series were obtained from the House Price Index Datasets of the Federal Housing Finance Agency (2023)

Two breaks were found in the case of West South Central, three in the case of East North Central and West North Central, four in the majority of cases, and five in the Pacific case. Many of the series exhibited breaks around April to June 2007, namely just before the U.S. sub-prime mortgage crisis. Breaks were also exhibited in April to August 2011 immediately before Operation Twist when the Fed restructured its debt portfolio by selling short-term T-bills and buying long-term debt with the aim of flattening the yield curve and boosting the mortgage market as well as other forms of credit. In addition, breaks were exhibited in May–June 2020 following the end of the shortest U.S. recession on record (caused by the coronavirus pandemic).

Table 5a-j displays the estimated coefficients for each series and each subsample. In the case of the East North Central series, the estimates of* d* are now much smaller than when considering the whole sample. Thus, the unit root null hypothesis cannot be rejected in the first three subsamples (with the data ending in May 2020). Only for the last subsample (June 2020—August 2022) was the estimate of *d* found to be much higher than one. The time trend is positive in the first, second and especially in the last subsample, being negative for the time period between May 2006 and October 2011.

**Table 5:** Estimates for each subsample

5a) EAST NORTH CENTRAL	d (95% band)	Intercept (t-value)	Time trend (t-value)
January 1991—April 2006	0.95 (0.90, 1.01)	99.463 (276.89)	0.5119 (24.52)
May 2006—October 2011	0.90 (0.77, 1.10)	193.605 (193.66)	-0.5820 (-5.94)
November 2011—May 2020	0.99 (0.90, 1.12)	157.294 (224.40)	0.7586 (11.45)
June 2020—August 2022	1.58 (1.00, 1.96)	233.831 (181.58)	3.0793 (2.63)
5b) EAST SOUTH CENTRAL	d (95% band)	Intercept (t-value)	Time trend (t-value)
January 1991- November 2004	0.78 (0.71, 0.87)	99.326 (229.70)	0.4240 (33.70)
December 2004 – April 2007	0.85 (0.65, 1.15)	172.238 (314.87)	0.9586 (14.40)
May 2007—October 2011	0.38 (0.16, 0.65)	200.725 (276.07)	–0.4421 (–19.25)
November 2011—May 2020	0.77 (0.71, 0.85)	177.178 (124.47)	0.8142 (14.40)
June 2020—August 2022	1.38 (0.96, 1.77)	261.502 (148.12)	3.6283 (3.54)
5c) MIDDLE ATLANTIC	d (95% band)	Intercept (t-value)	Time trend (t-value)
January 1991- June 1999	1.02 (0.93, 1.13)	99.873 (201.33)	0.1395 (2.54)
July 1999—May 2007	1.15 (1.07, 1.25)	113.332 (130.25)	1.0491 (6.26)
June 2007—February 2012	0.74 (0.62, 0.96)	218.626 (217.88)	–0.5227 (–9.31)
March 2012—May 2020	0.93 (0.86, 1.02)	190.441 (206.96)	0.6136 (8.85)
June 2020—August 2022	1.22 (0.37, 1.86)	248.996 (128.65)	3.1348 (4.38)
5d) NEW ENGLAND	d (95% band)	Intercept (t-value)	Time trend (t-value)
January 1991—January 1999	1.00 (0.91, 1.12)	99.846 (119.59)	0.1538 (1.81)
February 1999—November 2005	0.98 (0.89, 1.11)	114.514 (150.64)	1.4513 (18.72)
December 2005—January 2012	0.74 (0.62, 0.91)	232.421 (166.55)	–0.5459 (–8.51)
February 2012—May 2020	0.80 (0.72, 0.90)	192.446 (157.61)	0.7330 (13.28)
June 2020—August 2022	1.18 (0.59, 1.68)	264.817 (91.77)	3.8984 (4.12)
5e) MOUNTAIN	d (95% band)	Intercept (t-value)	Time trend (t-value)
January 1991—September 2003	1.01 (0.94, 1.08)	99.359 (206.70)	0.6591 (16.14)
October 2003—February 2007	1.30 (1.17, 1.47)	199.963 (169.95)	1.9967 (3.98)
March 2007—June 2011	1.07 (0.91, 1.31)	290.647 (140.34)	–1.7430 (–4.76)
July 2011—June 2020	0.87 (0.78, 1.00)	194.904 (127.57)	1.8698 (21.67)
July 2020—August 2022	1.94 (1.62, 2.24)	396.863 (150.99)	–
5f) PACIFIC	d (95% band)	Intercept (t-value)	Time trend (t-value)
January 1991—February 1997	0.92 (0.81, 1.08)	100.091 (299.37)	-0.0554 (-1.93)
March 1997—October 2003	1.25 (1.17, 1.34)	95.902 (162.03)	1.1386 (6.31)
November 2003—October 2006	1.55 (1.43, 1.72)	188.256 (153.80)	2.5562 (2.45)
November 2006—February 2012	1.42 (1.33, 1.55)	276.852 (161.52)	–
March 2012—May 2020	0.99 (0.89, 1.13)	170.539 (85.82)	1.5874 (8.28)
June 2020—August 2022	1.91 (1.65, 2.21)	171.434 (125.23)	–
5 g) SOUTH ATLANTIC	d (95% band)	Intercept (t-value)	Time trend (t-value)
January 1991—January 1998	0.83 (0.71, 1.01)	99.704 (314.63)	0.2359 (12.74)
February 1998—April 2007	1.38 (1.33, 1.46)	119.982 (208.65)	0.8504 (3.13)
May 2007—July 2011	1.02 (0.90, 1.19)	241.736 (134.07)	–1.3327 (–4.91)
August 2011—May 2020	0.86 (0.78, 0.97)	173,325 (179.51)	1.0935 (20.73)
June 2020—August 2022	1.73 (1.29, 2.13)	290.733 (151.35)	–
5 h) WEST NORTH CENTRAL	d (95% band)	Intercept (t-value)	Time trend (t-value)
January 1991—June 2007	1.08 (1.03, 1.15)	99.514 (196.79)	0.5614 (10.55)
July 2007—April 2011	0.64 (0.45, 0.89)	213.457 (166.87)	–0.5291 (–7.99)
May 2011—May 2020	0.80 (0.74, 0.87)	186.942 (181.16)	0.8551 (19.47)
June 2020—August 2022	1.37 (0.75, 1.83)	279.807 (158.86)	3.2519 (3.29)
5i) WEST SOUTH CENTRAL	d (95% band)	Intercept (t-value)	Time trend (t-value)
January 1991—July 2011	1.07 (1.02, 1.13)	99.704 (136.75)	0.3507 (5.28)
August 2011—June 2020	0.64 (0.56, 0.74)	186.911 (231.33)	1.0217 (50.09)
July 2020—August 2022	1.50 (0.99, 1.93)	300.953 (133.65)	3.4651 (1.97)
5j) USA	d (95% band)	Intercept (t-value)	Time trend (t-value)
January 1991—January 1998	1.07 (0.90, 1.32)	99.756 (498.92)	0.2508 (8.70)
February 1998—April 2007	1.47 (1.40, 1.56)	121.311 (389.67)	0.7091 (3.54)
May 2007—August 2011	–	–	–
September 2011—May 2020	1.16 (1.05, 1.33)	176.826 (338.61)	0.9780 (9.68)
June 2020—August 2022	2.22 (1.81, 2.64)	283.332 (228.63)	3.4094 (1.79)

In brackets are the 95% confidence bands in column 2, and the t-statistics for the estimated coefficients in columns 3 and 4. Convergence was not achieved in the case of the third sub-sample, probably due to the small number of observations. In all cases, the *p*-values were <0.005. The starting date is January 1991 and the final date is August 2022. The series were obtained from the House Price Index Datasets of the Federal Housing Finance Agency (2023)

Concerning the East-South-Central series, the values of *d* are now even smaller. The unit root hypothesis is rejected in favour of mean reversion (*d* < 1) during the first, third and fourth subsamples. It cannot be rejected during the second and the last subsamples. All the time trend coefficients are significant, being positive in all subsamples except the third one from May 2007 to October 2011. The highest coefficient on the time trend again corresponds to the last subsample.

As for the Middle Atlantic series, there are also four breaks and thus five subsamples. The estimates of *d* are between 0.74 (June 2007—February 2012) and 1.22 (June 2020—August 2022). As in the other cases, the time trends are all significantly positive, except the third one for the period starting in June 2007. Once again, the estimated time trend coefficient is significant and particularly high in the last subsample.

Very similar results were obtained for New England, though now mean reversion (i.e., significant evidence of *d* smaller than one) was found for the third and four subsamples (December 2005—January 2012, February 2012—May 2020) and a negative trend for the third subsample (December 2005—January 2012). The positive trend coefficients are equal to 0.1538 for the first subsample; 1.4513 for the second subsample; 0.7330 for the fourth subsample, and 3.8984 for the final subsample starting in June 2020.

In the case of the Mountain series, the results are slightly different. Mean reversion was not found in any single case, and *d* is statistically higher than one in the second and last subsamples, in the latter case being insignificant. Five breaks were detected in the case of the Pacific series. Mean reversion did not occur in any subsample, and *d* was estimated to be much higher than one, especially in the last subsample. The time trend is negative in the first subsample, positive in the second, third and fifth, and insignificant in the fourth and sixth.

Regarding the South Atlantic series, breaks were detected in January 1998, April 2007, July 2011, and May 2020. Mean reversion occurred in the fourth subsample (from August 2011 to May 2022) and the time trend was insignificant in the last subsample.

In the case of the West North Central series, mean reversion took place in the second (July 2007—April 2011) and third (May 2011—May 2020) subsamples, with a significant negative trend in the former. There were only two breaks (July 2011 and June 2020) in the West-South-Central series. Mean reversion occurred in the second subsample, and the time trend is significantly positive in all three subsamples.

Finally, there were four breaks in the U.S. aggregate series (January 1998, April 2007, August 2011 and May 2020), and no mean reversion in any single case. The time trend coefficients are all positive, although convergence could not be achieved for the third subsample (May 2007—August 2011), probably as a result of the small number of observations. In the other cases, the time trend coefficient was significantly positive, again being particularly high in the last subsample.

## Conclusions

This paper uses fractional integration methods to analyse the behaviour of U.S. house prices, more specifically the monthly Federal Housing Finance Agency (FHFA) House Price Index for census divisions and the U.S. as a whole, over the period from January 1991 to August 2022. The full sample estimates imply that the order of integration of the series is above one in all cases, and is particularly high for the aggregate series. However, when the possibility of structural breaks is taken into account, segmented trends are detected. The subsample estimates of the fractional differencing parameter tend to be lower, with mean reversion occurring in a number of cases, and the time trend coefficient being at its highest in the last subsample, which in most cases started around May 2020.

On the whole, it is clear that there is heterogeneity between housing markets in different geographical areas of the U.S., which might reflect differences in the number of buyers and sellers in each case as well as other local factors. These cannot be captured by the aggregate series. Thus, it is important to obtain evidence for the various census divisions as well. In particular, the individual series were found to be less persistent than the aggregate one, and also to be subject to structural change. The detected breaks appeared to correspond to well-known economic and policy developments (such as the sub-prime mortgage crisis, changes in the Fed’s debt portfolio, and the rebound after the early stages of the coronavirus pandemic). House price persistence is transmitted to other macroeconomic and financial variables, Therefore, accurate information on persistence is crucial for policy decisions, with different policy measures needed in response to shocks depending on the degree of persistence (Himmelberg et al., 2005). The present study offers thorough evidence on this property for U.S. house prices in different geographical areas and time periods, and thus has important policy implications, in particular for crisis management and/or prevention.

The present study has some limitations. In particular, the univariate nature of the chosen method does not allow for possible correlations between the various series. This would require estimating a panel model and is beyond the scope of the current analysis, which focuses instead on the stochastic properties of the individual series. Spatial econometrics would be an alternative approach, though totally unrelated to long-memory models. It would also be interesting to distinguish between prices for different types of houses, or to convert all of them to equivalent houses and then analyse the prices. This is left for subsequent studies. Future research could also allow for non-linearities in house prices. One possible approach would be based on Chebyshev’s polynomials (Bierens, 1997), which do not produce abrupt changes in the series (unlike models with structural breaks), and can easily be used in the context of fractional integration. An alternative framework would include non-linear (deterministic) trends based on Fourier functions in time or neural networks, still within the long-memory framework.

## Data Availability

Data used in this work are available from the authors upon request.
